# Pullulan-based dissolving microneedle arrays for enhanced transdermal delivery of small and large biomolecules

**DOI:** 10.1016/j.ijbiomac.2019.12.184

**Published:** 2020-03-01

**Authors:** Lalitkumar K. Vora, Aaron J. Courtenay, Ismaiel A. Tekko, Eneko Larrañeta, Ryan F. Donnelly

**Affiliations:** a School of Pharmacy, Queen's University Belfast, Medical Biology Centre, 97 Lisburn Road, Belfast BT9 7BL, UK; b Department of Pharmaceutics and Pharmaceutical technology, Faculty of Pharmacy, Aleppo University, Syria

## Abstract

One specific technological advance in transdermal drug delivery is the development of dissolving microneedles (DMNs), which efficiently deliver therapeutics through a rapid dissolution of polymers after penetration into the skin. However, there is a limited range of water soluble, biodegradable polymers that can be used to manufacture DMN. Here, we report for the first time, the preparation and characterisation of a DMN system from the carbohydrate biopolymer, pullulan (PL). PL gels, of varying concentration, were studied for viscosity, film formation properties, and subsequently, microneedle formation. Model molecules and protein/peptide were loaded into PL DMN and characterised. The stability of model biomolecules, such as FITC-BSA and insulin, following DMN manufacture were assessed using circular dichroism. Ex-vivo porcine skin permeation studies using Franz diffusion cell apparatus for Flu-Na and FITC-BSA loaded PL-DMN were conducted. This study demonstrates that PL DMNs may serve as a promising tool for efficient transdermal drug delivery.

## Highlights

• Pullulan microneedles deliver both high and low molecular weight molecules across skin in vitro.
• Biomolecules were stable in gels used to prepare pullulan microneedles.
• Pulluan microneedles exhibited promising physical properties.
• Pullulan microneedles may be useful in transdermal delivery of biomolecules.

## Graphical abstract

**Figure:**
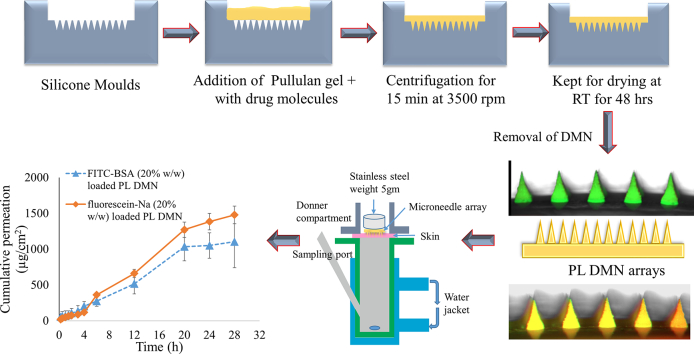

## 1 Introduction

Transdermal drug delivery systems (TDDS) are well-known within the pharmaceutical domain since the invention of nitroglycerin ointment [1]. Transdermal administration is a favoured route of administration as it provides advantages such as improved dosage efficacy with controlled or prolonged drug delivery, enhanced patient compliance, and reduced systemic side effects [2]. Importantly, however, the outermost barrier of the skin, the *stratum corneum* (*SC*), allows only low-molecular-mass lipophilic drugs to passively penetrate the skin. This *SC* barrier considerably limits the transdermal delivery of larger molecules such as proteins and therapeutic genes. To increase the permeability of the *SC*, techniques such as ultrasound, iontophoresis, electroporation, thermal ablation and microdermabrasion have been developed. However, most of these techniques have not progressed past the pre-clinical phase of testing due to the risk of skin irritation, which is unacceptable in a clinical setting [3]. Permeation enhancer and prodrug approaches have also been explored extensively for TDDS with limited success [4].

To overcome the challenges associated with current technologies, microneedle (MN) arrays are currently of significant interest in both transdermal drug delivery and transdermal diagnostics. MNs are minimally invasive devices consisting of numerous micron-sized projections arranged on a baseplate. The MNs perforate the SC, forming temporary aqueous micropores through which drugs can diffuse to the dermal microcirculation. However, with an average height of 400–900 μm, MNs are short enough to avoid stimulation of dermal nerves and do not induce bleeding [5,6].

First-generation MNs, made from silicon, metals or glass, were designed to create the pore in the skin into which the drug or vaccine diffused. The disadvantages of these types of MNs are; limited loading dose of drugs, and the critical risk that MNs can be accidentally broken and therefore remain in the skin for a long period of time. Consequently, microneedle arrays which are fabricated from biodegradable [7] or dissolving polymers, have recently received considerable attention [8]. Dissolving MN (DMN) arrays are fabricated to incorporate drug molecules into a soluble polymeric matrix and the drugs can be released in the skin upon dissolving the inserted MNs. The advantage of this new kind of DMNs is that they can be formulated more cost-effectively compared to silicon or metal MNs. Neither do they pose a safety concern as the sharp tips fully dissolve in skin interstitial fluid to release their drug payload. Unlike coated metal or silicon MNs, drugs can be encapsulated within the matrix of polymeric MNs, dramatically increasing their drug loading capacity [9]. Using different polymers that display different degradation profiles, swelling properties and responses to biological/physical stimuli can allow for the development of customisable drug delivery systems. Moreover, the use of DMN can effectively extend the shelf-life of proteins/peptides, and lower the cost of cold chain processes compared to their conventional solution-based counterparts [10].

However, there is a limited range of biodegradable polymers that can be used to manufacture MNs. Addition to that, water-soluble polymers generally have weaker mechanical strength compared to non-dissolving materials like silicon or metal, and drug encapsulation may further weaken their biomechanical strength [11,12]. Thus, appropriate geometry design and material selection are particularly important for dissolvable MNs fabrication. To date, dissolving MNs have been fabricated from various materials, such as sugars, galactose [12], carboxymethylcellulose [13], poly(vinylpyrrolidone) [14,35], poly (methyl vinyl ether-maleic acid) [15], chondroitin sulfate [16] sodium hyaluronate (HA) [17] and Poly(vinyl alcohol) [31]. Nevertheless, we reported the serious processing, handling and mechanical stability issues with carbohydrate and sugar-based MNs [12]. Considering the limitations of explored polymeric materials for MN drug delivery, the film-forming carbohydrate biopolymer, pullulan was investigated in this current work to fabricate the self-dissolving MN for the delivery of model small and large biomolecules.

Pullulan (PL) is a natural polymer produced by the yeast like fungus *Aureobasidium pullulans* in starch and sugar cultures. PL is a hydrophilic linear biopolymer mainly consisting of maltotriose units interconnected α-(1,6) glycoside bonds. This unique linkage pattern provides pullulan with exceptional physical properties to form film that is mechanically strong, transparent, water-soluble and with low permeability to oil and oxygen [18]. Addition to that, there is no toxicity or mutagenicity [19]. PL is easy to chemically modify because it has more numbers of hydroxyl groups. Owing to these peculiar characteristics, PL and PL derivatives hold a vital role in numerous biomedical applications such as tissue engineering, oral films, capsule coating, vaccination, targeted drug and gene delivery [18,20]. Therefore, high hydrophilicity, good mechanical strength and biocompatibility make PL an excellent candidate to manufacture the dissolving MN. This is the first study to prove the use of carbohydrate polymer PL, for dissolving MNs patches for transdermal delivery.

The aim of this study was to develop DMN from the carbohydrate biopolymer PL for transdermal delivery of small molecule drugs and biomolecules. The first objective was to investigate the rheological and mechanical properties of PL to assess the required formulation characteristics for DMN fabrication. The mechanical strength of drug-free DMN was evaluated, and the insertion capability in neonatal porcine skin was investigated. Following this, PL DMN formulations were developed and characterised with model small molecules (methylene blue and fluorescein sodium) and with large protein/peptide biomolecules (FITC-BSA and insulin).

## 2 Materials and methods

### 2.1 Materials

Phosphate buffered saline (PBS), fluorescein sodium, methylene blue, fluorescein isothiocyanate labelled bovine serum albumin (FITC-BSA), insulin (human recombinant, produced by recombinant DNA technology in yeast *Saccharomyces cerevisiae*, >27.5 IU/mg) were purchased from Sigma Aldrich Co. (St. Louis, MO, USA). Double distilled ionized water was prepared using NanoPure Infinity Ultrapure water purification system. Pullulan (viscosity: 133 mm^2^/s, 10%w/w, Ubbelohde type viscometer, average Mw 200 kDa) was kindly provided by Hayashibara Ltd. (Japan). Parafilm® M (~127 μm thickness), made of olefin-type materials, was purchased from Brand GMBH (Wertheim, Germany). All other chemicals used were of analytical reagent grade.

### 2.2 Rheological study of PL gel

Rheological characterisation of different concentrations of PL gel was performed using a TA instruments AR 1500 Rheometer (TA instruments, Elstree, Herts, UK) fitted with a 40 mm diameter steel parallel plate. Flow rheology testing was conducted at continuous controlled ramp mode with the shear rate increased from 0 to 50/s and 25 °C. Viscosity was determined by the application of the Power law.

### 2.3 Preparation and characterisation of PL film

PL films were prepared by casting the different concentrations of aqueous gels of PL (10%, 15% 20% and 25% w/w). Each solution was added to an individual mould consisting of a stainless steel frame secured on 5 cm × 2 cm Perspex® base. To allowing even spreading of the solution, the mould was placed on a flat surface and dried at room temperature for 48 h. Film thickness was measured by using a digital micrometer (Hilka, ProCraft, Surrey, UK). A TA-XT2 Texture Analyser (Stable Microsystems, Haslemere, UK) was used to conduct tensile strength using PL film strips (50 mm × 5 mm). The strips were secured between two grips set at 20 mm spacing and a cross-head speed of 0.5 mm/s was used.

### 2.4 Fabrication of plain PL DMN

**Fig. 1:**
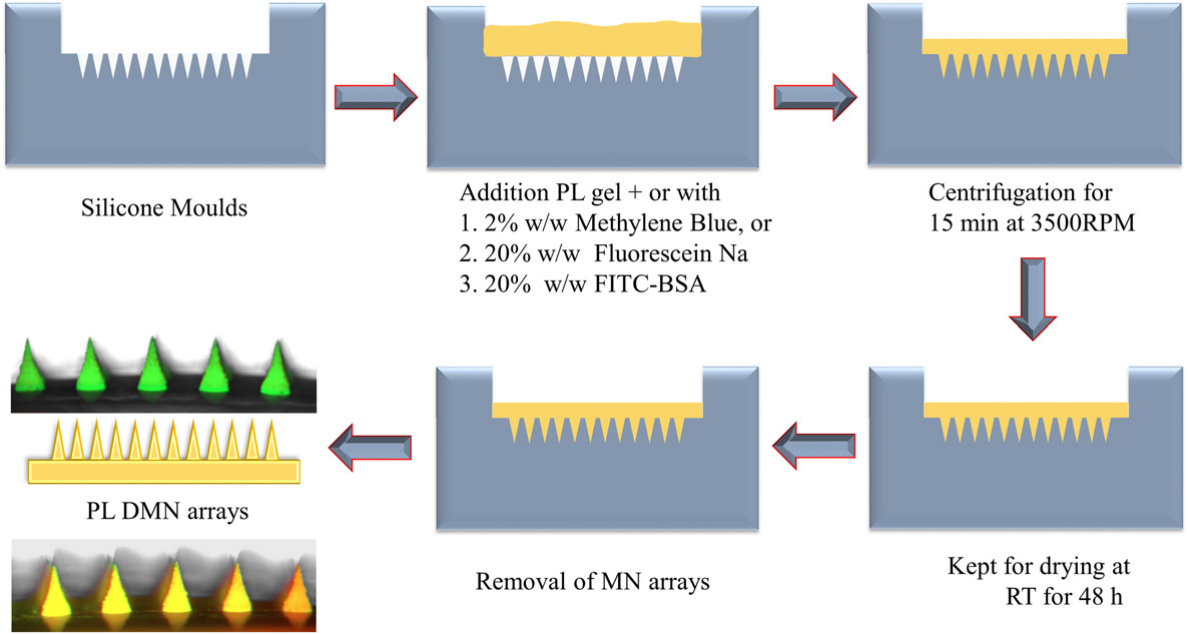
Schematic representation of fabrication of PL and drug-loaded PL DMN arrays.

PL DMN arrays were fabricated from aqueous solutions of PL (10%, 15% 20% and 25% w/w) as summarized in Fig. 1. Approximately 500 mg of blend was poured into laser-engineered silicone conical 19 × 19 (19 × 19 needle array density per 0.49 cm^2^ area, 600 μm needle height, 300 μm needle width at the base, and 150 μm interspacing) and 12 × 12 arrays (12 × 12 needle array density per 0.49 cm^2^ area, 600 μm needle height, 300 μm needle width at the base, and 150 μm interspacing) micromould templates. The filled moulds were centrifuged at 2205 ×*g* for 15 min. Finally, the arrays were dried at room temperature for 48 h and they were stored at room temperature in a desiccator prior to the analysis. Drying time and temperature parameters for PL (20% w/w) DMN arrays were assessed at room temperature, 40 °C and 80 °C for 48 h, 24 h and 12 h respectively.

### 2.5 Fabrication of drug-loaded PL DMN

Methylene blue (MB, 2% w/w) and fluorescein sodium (Flu—Na, 20% w/w), as a water-soluble blue (absorption wavelength of 664 nm) and red dye (absorption wavelength of 450 nm) respectively, were mixed into the PL (20% w/w) solutions and used as a model low molecular weight drugs. BSA-FITC (20% w/w), as a high molecular weight model protein with an excitation/emission wavelength of 480/520 nm, was dissolved in the PL solutions as well. Another model biomolecule, insulin (20% w/w), was dissolved in a 0.1 M HCl solution and then added to the PL solutions. These model drugs loaded PL DMN were prepared as per the described process in Section 2.4 and Fig. 1. The morphology of drug-loaded DMN patches was investigated using a Keyence VHX700F Digital Microscope (Keyence, Osaka, Japan). In addition, scanning electron microscopy (TM3030 benchtop SEM, Hitachi, Krefeld, Germany) was used for high-resolution imaging. The instrument was operated at 15 kV and images were captured at magnifications between 40 and 250×.

### 2.6 Mechanical and insertion properties of PL DMN

Mechanical tests were used to assess compression and insertion properties of blank PL DMN and MB-loaded DMN arrays. These tests were conducted using a TA-XT2 Texture Analyser in compression mode, as reported previously [21]. Briefly, to test compression, DMN arrays were first visualized using a stereomicroscope to determine the initial needle height. DMN arrays were then attached to the moveable cylindrical probe of the Texture Analyser using double-sided adhesive tape and pressed by the test station against a flat aluminium block at a rate of 0.5 mm/s for 30 s and a force of 0.36 N per DMN applied. Pre-test and post-test speeds were set at 1 mm/s, and the trigger force was set at 0.049 N. DMN heights were measured again using the stereomicroscope and percentage reduction in height, following the application of the axial compression load, calculated.

Insertion properties of the DMN were investigated using Parafilm M® (PF), a flexible thermoplastic sheet made of olefin-type material, which has recently been highlighted as a useful skin simulant for insertion of MNs [22]. The initial heights of the DMNs were measured microscopically prior to the test. The PF sheet was folded into an eight-layer film (~1 mm thickness). Following attachment of the MN array to the movable probe of the Texture Analyser, the probe was lowered onto the folded PF at a speed of 1.19 mm/s until the required force of 32 N was exerted. The force was held for 30 s. The MN arrays were then removed from the polymeric sheet and the final MN heights measured, allowing the percentage reduction on insertion to be calculated. The PF sheet was unfolded and the number of holes in each layer evaluated using the stereomicroscope. The percentage of holes created versus depth of PF was plotted [22].

### 2.7 Determination of water content of PL DMN

The percentage water content of PL arrays was determined with a Q500 Thermo Gravimetric Analyser (TA Instruments, Elstree, Herts, UK). Samples of 10.0 mg were heated from ambient temperature to 300 °C at a heating rate of 10 °C min^−1^. Nitrogen flow rates of 40 ml min^−1^ (balance purge gas) and 60 ml min^−1^ (sample purge gas) were maintained for all samples. The data from thermogravimetric analysis experiments were analyzed with TA Instruments Universal Analysis 2000 software, version 4.4A (TA Instruments, Elstree, Herts, UK).

### 2.8 Dissolution studies

The dissolution of the PL DMNs was evaluated in-situ using full-thickness neonatal porcine skin. The skin was obtained from stillborn piglets and excised within 24 h of birth using an electric dermatome (Integra Life Sciences™, Padgett Instruments, NJ, USA). The skin was then wrapped in aluminium foil and stored at −20 °C until use. Before performing penetration studies, the skin was carefully shaved using a disposable razor and washed with phosphate-buffered saline (PBS, pH 7.4). Skin samples were then placed on top of dental wax, to give the skin support and the underside of the skin bathed in PBS (pH 7.4) at 37 °C for 30 min to equilibrate. DMN arrays were then inserted into the centre of the skin section using manual pressure and a circular steel weight placed on top to ensure the array remained in place. MN arrays were removed at various time points and immediately viewed under a Leica EZ4 D stereomicroscope (Leica Microsystems, Milton Keynes, UK).

### 2.9 Optical coherence tomography

Optical coherence tomography (OCT) was used to visualize the insertion of PL DMN into full-thickness porcine skin. DMN patches were applied with an application force of 32 N per patch by Texture Analyser for 30 s. OCT was employed with a laser centre wavelength of 1305.0 ± 15.0 nm to facilitate real-time high-resolution imaging of upper skin layers. The skin was scanned at a frame rate of up to 15 B-scans (2D cross-sectional scans) per second (scan width = 2.0 mm). The 2D images were examined using the imaging software ImageJ® (National Institute of Health, Bethesda, USA). The scale of image files obtained was 1.0 pixel = 4.2 μm, thus allowing accurate measurements of the depth of MN penetration, the distance between the MN baseplate and the *SC* [23]. OCT images were captured immediately upon insertion and then after 10 min post-insertion.

### 2.10 Insertion of MB loaded PL DMN arrays into excised neonatal porcine skin

In this study, dermatomed neonatal porcine skin was used as a model of the human skin because the structure of porcine skin is similar to that of human skin [24]. Skin (approximately 350 μm thickness) was obtained from stillborn piglets and excised within 24 h of birth using an electric dermatome. The skin was then wrapped in aluminium foil and stored at −20 °C until use. Before performing penetration studies, the skin was carefully shaved using a disposable razor and washed with phosphate-buffered saline (PBS, pH 7.4). The skin surface was dried using tissue paper and placed dermis side down on a dental wax sheet (Anutex®, Kemdent Works, Swindon, UK). MB (2% w/w) loaded PL (20% w/w) DMN arrays were applied perpendicularly into the skin using manual pressure for 30 s. Cylindrical 5.0 g stainless steel weights were placed on top of the array to hold them in place and incubated for 5 min, 6 h and 24 h at 37 °C. Afterwards, the DMN was removed and the skin was visualized using the digital microscope. The number of holes created was then counted and the percentage of holes created was recorded.

### 2.11 Circular dichroism analysis of FITC-BSA and insulin loaded PL DMN arrays

Samples of insulin, FITC-BSA, and biomolecules-loaded PL DMN formulations were analyzed by circular dichroism (CD) to assess secondary structure pre- and post- formulation. FITC-BSA and insulin loaded PL DMN were dissolved in PBS (pH 7.4) and submitted to CD measurement (Jasco J-810, Easton, MD). CD spectra of free FITC-BSA and insulin were also obtained by using quartz cuvettes (0.5 ml volume, 1 mm length) on a JASCO J-810 spectrometer equipped with a cell compartment thermo-regulated at 25 °C with wavelength range 190 to 290 nm.

### 2.12 Ex-vivo drug delivery from flu-Na and FITC-BSA-loaded dissolving PL DMN arrays

The permeation of Flu-Na and FITC-BSA from PL DMN arrays across dermatomed (~ 350 μm) neonatal porcine skin was investigated using modified Franz diffusion cells, as described previously [25]. The drug-loaded PL DMN array was placed on top of the dermatomed porcine skin and inserted using manual pressure for 30 s. A 5.0 g cylindrical, stainless steel weight was placed on top of the inserted array. The donor compartment of the apparatus was clamped onto the receiver compartment. The donor compartment and sampling arm were sealed using Parafilm M®. The receiver compartment contained PBS (pH 7.4) was thermo-regulated at 37 ± 1 °C. Syringes (1.0 ml) with 8.0 cm sampling needles were used to remove 200 μl of the Franz cell contents at appropriate time points and 200 μl of pre-warmed PBS was subsequently added to replace this. The concentration of the released Flu-Na and FITC-BSA in the receiver compartment was determined using UV–vis and fluorescence spectroscopy (FLUOstar® Omega, BMG labtech ltd, Offenburg, Germany) respectively.

### 2.13 Statistical analysis

All quantitative data were expressed as means ± SD from triplicate measurements unless otherwise noted. Differences between groups were assessed for significance using an unpaired, two-tailed Student's *t*-test. The threshold for significance was *p* < 0.05. Statistical analysis was performed using Prism 6 (GraphPad Software, USA).

## 3 Result and discussion

### 3.1 Characterisation of PL gel and PL films prepared from different concentration

**Fig. 2:**
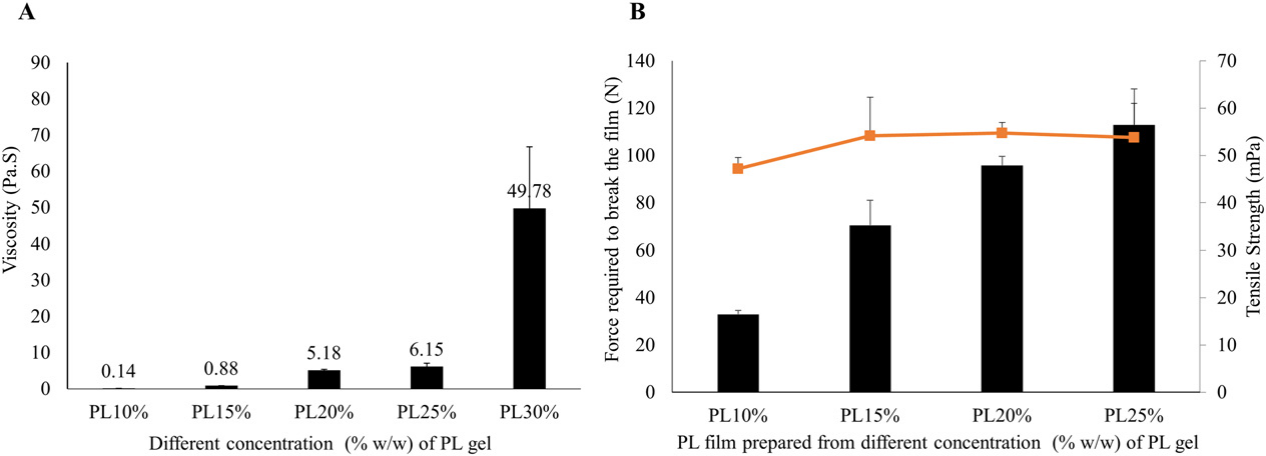
(A) Viscosity measurements of PL gel (10%, 15%, 20%, 25% and 30% w/w) and (B) force required to break the film with a tensile strength that prepared from different concentration of PL gel (Means ± SD, *n* = 3).

The viscosity of the aqueous PL formulations is a crucial parameter as PL gel should be able to fill the cavities of MN silicone moulds when positive pressure or centrifugation forces are applied during the DMN manufacturing. However, in order to prepare MNs with good mechanical strength and also avoid shrinkage of the DMN arrays during the drying stage, it is important to use gels with sufficiently high polymer concentrations. The evolution of the viscosity vs. different concentration of PL gel is shown in Fig. 2A. Below 25% w/w concentration, PL exhibited newtonian behaviour. These formulations presented viscosities ranging from 0.1 to 6 Pa∙s. However, when the PL concentration reached 30% w/w the viscosity increased significantly (*p* < 0.001) and the gel formulations displayed pseudoplastic behaviour. Therefore, only formulations containing up to 25% w/w of PL is processable for DMN formulation development.

The film-forming polymer with good mechanical strength is required to produce the DMN. Therefore, the analysis of mechanical properties of the polymeric film is preliminary step for the DMN development. Fig. 2B shows that the force required to break the PL film is directly proportional to the initial polymer concentration. Tensile strength of PL films was found to be 52.4 ± 3.5 N/mm^2^.

### 3.2 Optimisation of plain PL DMN

**Fig. 3:**
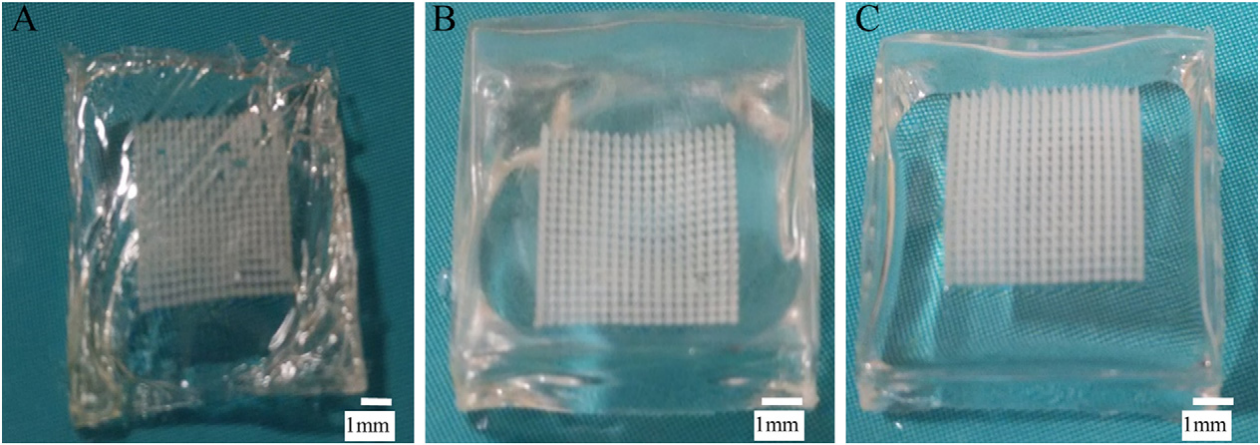
PL (20% w/w) DMN arrays prepared and dried at different temperatures; (A) 80 °C (B) 40 °C (C) Room temperature.

PL DMN (20% w/w PL) was dried at higher temperatures as a way to reduce drying time, however, the DMN baseplate surface became uneven (Fig. 3). In comparison, DMN arrays prepared at room temperature (25 °C), examined microscopically, displayed well-formed DMNs with sharp tips, a complete array of needles.

**Fig. 4:**
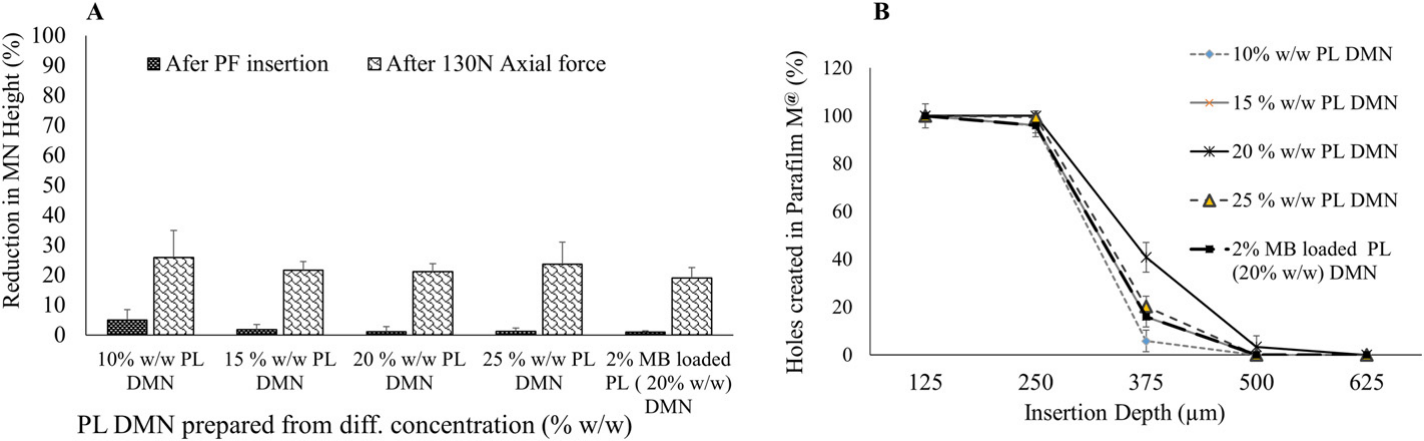
Mechanical characterisation PL DMN prepared from different concentration and MB loaded PL MN arrays; (A) Percentage reduction in the height of the needles after different compression force applied (means ± SD, *n* ≥ 3) and (B) Percentage of holes created in each PF layer after the application of PL DMN arrays with a 0.089 N per needle insertion force (means ± SD, n = 3).

The mechanical properties of DMNs should be evaluated to determine whether DMNs are strong enough to penetrate the skin and do not break during skin penetration. Mechanical properties of PL MNs prepared using the different concentrations of PL were evaluated as described previously by McCrudden et al. [25]. MN arrays were compressed against a metal block. As applied force was increased, there was a decrease in MN height but, particularly, none of the MN fractured, rather they became slightly compressed. However, this method does not represent the real insertion situation as skin is softer and more flexible. MN prepared using 20% w/w PL showed <21% in height reduction when compressed using 130 N force per MN patch (Fig. 4A). It was found to be significant difference (*P* < 0.05) in height reduction from 10% w/w PL MN compared to rest of PL MN after PF insertion. There were no statistically significant (*P* > 0.05) differences in % reductions in height from 15%, 20% and 25% w/w concentration of PL during compression and insertion tests. Considering that human typically applies 32 N when manually applying MNs, this test can be used as quality control [22]. In order to evaluate the insertion of the arrays, a skin simulant (Parafilm M®) was used following the method described by Larrañeta et al. [22]. After application of PL DMN array at 32 N force by Texture Analyser, the number of holes created in each layer of the PF sheet was assessed using a light microscope. Fig. 4B shows the percentage of holes created in each PF layer after applying PL DMN arrays. All PL DMN arrays prepared from different PL concentration showed similar insertion profiles without any significance difference (*P* > 0.05). Based good mechanical and insertion profile of PL film and DMN, PL DMN prepared from 20% w/w PL gel was taken further. The MB (2% w/w) loaded PL (20% w/w) DMN arrays showed similar height reduction at different forces compared to blank PL (20% w/w) DMN arrays. The insertion plot (Fig. 4) shows that the DMN arrays could reach an insertion depth of approximately 375 μm. This means that the application of DMN arrays with an insertion force of 0.089 N per needle for 30 s may be suitable for penetrating the skin. Most of the reported dissolving MN from the synthetic polymers were required to have combination of two or more polymers or additional plasticizer to get the enough mechanical strength [17]. While, in this work, PL alone is sufficient to get the required mechanical strength for insertion.

The water content of DMN is known to impact on mechanical properties including rigidity, flexibility, and dissolution of DMN. The high content of moisture will weaken MNs ability to penetrate the skin. The water content of plain PL DMN arrays was determined using thermogravimetric analysis. Bound and free water in PL DMN were 4 ± 0.3% and 2.3 ± 0.76%, respectively. In general, the total water content of the PL DMN arrays was <7% and this is lower than other polymeric DMNs reported [14,25,26]. High levels of water content can affect the stability of drug molecules; however, this is particularly important for biologics that are highly sensitive to moisture. Even though these DMNs were fabricated under normal lab conditions, manufacture of these DMNs in a controlled temperature and humidity could further decrease the moisture content of PL DMN thereby enhancing the storage stability of biomolecules. Nevertheless, at this water content level, we found that the PL DMNs preserved their mechanical strength, the stability of biomolecules and effectively penetrated the porcine skin tissue.

**Fig. 5:**
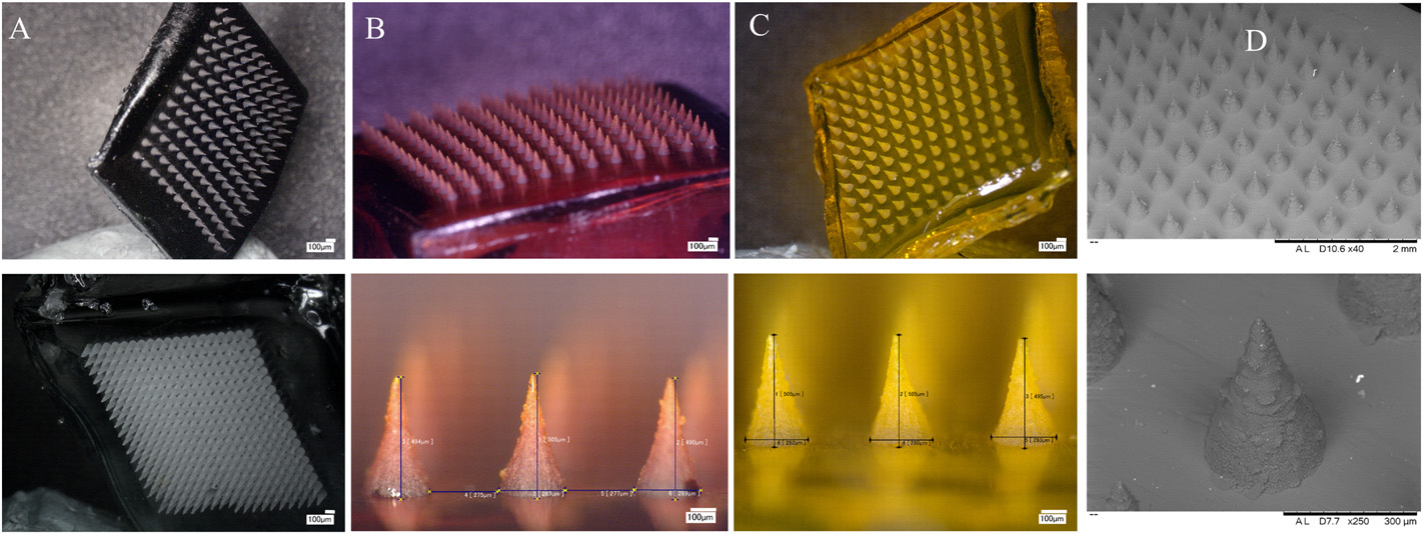
Digital microscopic images of different molecules loaded PL DMNs: (A) 2% w/w MB loaded PL DMN (12 × 12 and 19 × 19), (B) 20% w/w Flu-Na loaded PL DMN (12 × 12), (C) 20% w/w FITC-BSA loaded PL DMN (12 × 12) and (E) Electron microscopic image of PL DMN.

Different drug molecules loaded PL DMNs that appeared to be fully formed upon visual examination under a light microscope and were taken for further characterisation. Irrespective of the drug molecules and content (2% w/w MB, 20% w/w Flu Na and FITC-BSA) used in preparing the DMN, they each displayed reproducible MN formation with sharp tips. This is shown in Fig. 5 A–C with representative SEM images of 2% w/w MB DMN displayed in Fig. 5D.

### 3.3 Dissolution of PL DMN after insertion into porcine skin

**Fig. 6:**
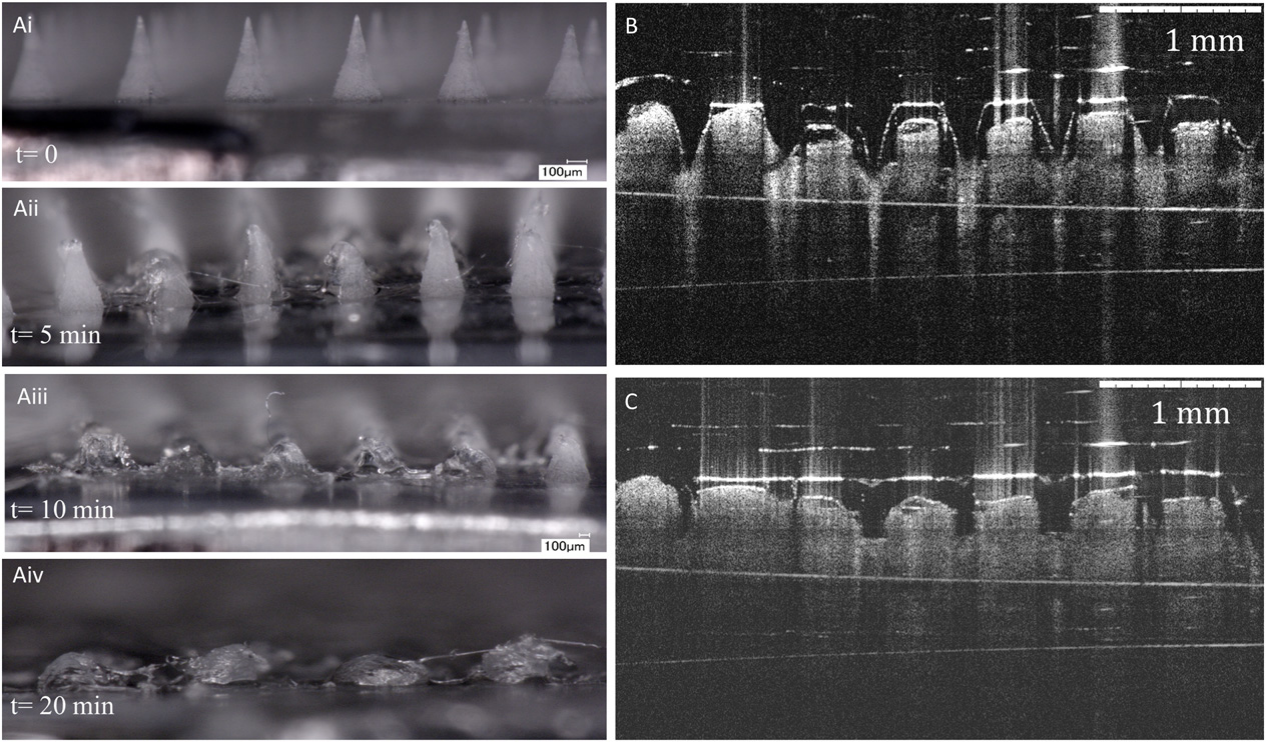
(A) Representative digital micrographs of the dissolution of blank PL DMN arrays (prepared from 20% w/w PL gel) at specific time points (T0: 0 min; T5: 5 min; T10: 10 min etc.) over a 30 min period following insertion into, and removal from excised neonatal porcine skin, (B) OCT images of DMN insertion in full-thickness porcine skin 12 × 12 (500 μm) and (C) Microchannel pores generated by 12 × 12 (500 μm).

Dissolution studies of the PL DMN formulations were undertaken with a view to predicting the time required for dissolution after inserting into full-thickness porcine skin. The DMN array formulated from PL showed dissolution of approximately 80% of needles height after 10 min. Despite the high in molecular weight of PL (~200 KDa) compared to the reported DMN with low molecular synthetic polymers [27], complete dissolution of PL DMN arrays within 20 min as displayed in Fig. 6Aiv. This could be useful for fast dissolving MN formulation development compared to non-biodegradable synthetic polymers.

### 3.4 Optical coherence tomography

To take real-time images of the PL DMN insertion into the porcine skin, a non-invasive optical imaging technique, OCT, was used in this study and the images are displayed in Fig. 6B and C. The inserted part of MN was calculated by measuring the non-inserted part of MN arrays by analyzing the distance between the baseplate and skin surface by ImageJ software. It was found that 403 ± 35.8 μm inserted out of total height of 504 ± 6.4 μm which is 80 ± 7.2% insertion. As depicted in Fig. 6C, the visible depth of microchannel pores created by PL DMN in full-thickness skin was 239.4 ± 8.4 μm. Moreover, the width of the created pores was measured as 160 ± 32.6 μm, as determined from the side view by the OCT analysis. These set of results showed that PL DMN were mechanically strong enough to insert into the porcine skin and further dissolve beneath the *SC*.

### 3.5 Insertion of MB loaded PL DMN arrays into excised neonatal porcine skin

**Fig. 7:**
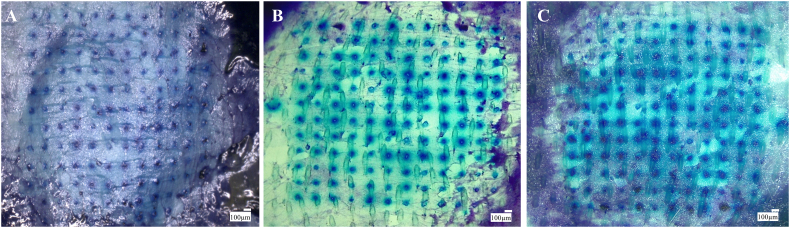
Digital images of MB deposited holes on neonatal porcine skin after insertion of MB (2% w/w) loaded PL (20% w/w) DMN arrays at different time points: (A) 5 min, (B) 6 h and (C) 24 h at 37 °C.

For efficient intradermal delivery, it is important that MNs penetrate into the skin and subsequently deposit their payload. The penetration efficiency of DMN was quantified as a percentage of the number of MNs penetrating into the epidermis of ex vivo porcine skin after the application of the MB loaded PL DMN array for 5 min, 6 h and 24 h. Fig. 7 shows top view of MB deposited holes in porcine skin after MB loaded PL DMN penetration at different time points. It was found that 95–100% of MNs penetrated into the porcine skin and this is comparable to results achieved with solid MNs [28]. These results indicate that PL DMN patches provide a sufficient capability of penetration via SC to create microholes, and deposit their payload after the DMN patch is peeled off at different time points.

### 3.6 Circular dichroism analysis of FITC-BSA and insulin loaded PL DMN arrays

**Fig. 8:**
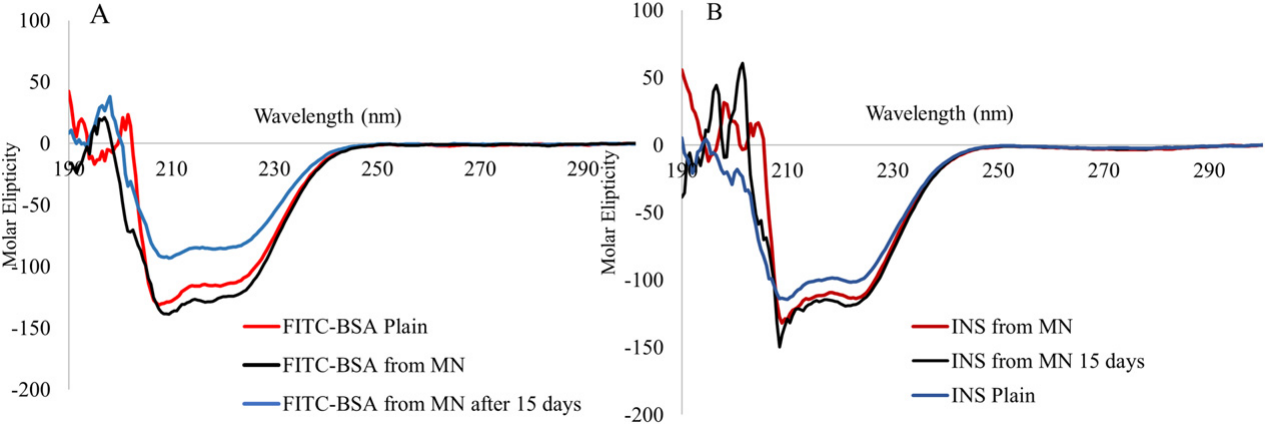
Stability of secondary structure of FITC-BSA and insulin (INS) by Circular Dichroism from PL DMN.

To evaluate the structural integrity of encapsulated BSA-FITC and insulin in PL DMN, we used CD spectroscopy, which is widely applied to examining the secondary conformation of proteins [29]. Insulin and BSA are most commonly used model biomolecules in novel drug delivery [34]. We found that the CD spectra of PL DMN loaded FITC-BSA and insulin were practically indistinguishable from that of native FITC-BSA and insulin solution (Fig. 8), indicating that the structural integrity of both proteins in PL DMN are preserved intact. The CD spectra of standard solutions of insulin and FITC-BSA exhibit a strong negative minimum at 208 nm and a lesser pronounced minimum at 222 nm. These bands are indicative of a typical α-helical structure. Both biomolecules were found to be stable following 15-day storage of MN arrays under ambient conditions. The fabrication of PL DMN does not require high temperatures. Furthermore, the solid-state matrix of non-ionic PL polymer contributes to enhanced storage stability by preventing the aggregation of biomolecules at room temperature [30].

### 3.7 Ex-vivo release of drug-loaded PL DMN in porcine skin

**Fig. 9:**
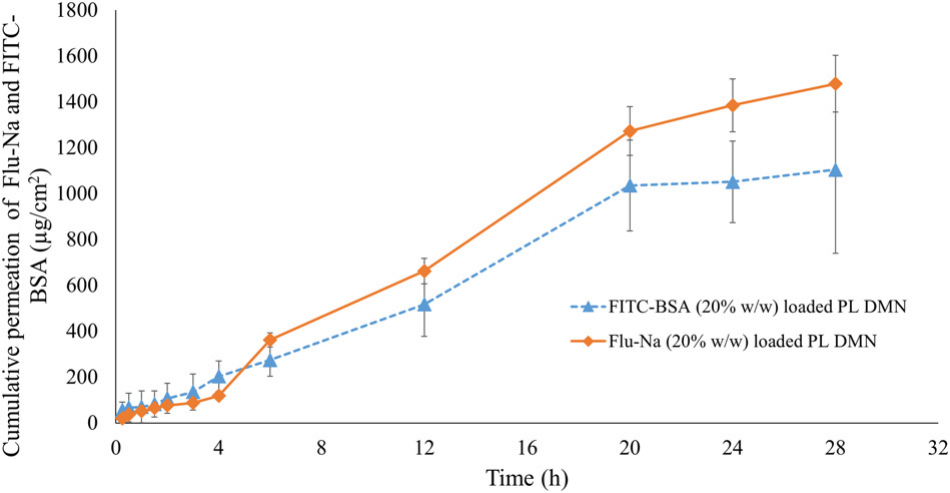
The ex-vivo cumulative permeation profile of FITC-BSA and Flu-Na across dermatomed (~350 μm) neonatal porcine skin when delivered using PL DMN arrays (means ± S.D., n ≥ 3).

This study was conceived as a proof of concept and the main objective was to explore the possibility of delivering the model biomolecules using PL DMNs first time. The model molecule-loaded PL arrays were manufactured containing drug in the needle tips and in a small baseplate. In this manner biomolecules can be delivered from the needle tips and consequently from the baseplate. The drug confined in the baseplate can permeate through the created pores [25]. Here, to study the permeation profiles of a small molecule, Flu-Na and a large protein, BSA-FITC, PL DMN patches were inserted into porcine skin and the drug release was monitored for 28 h. Fig. 9 illustrates the permeation profiles of FITC-BSA and Flu-Na released from PL DMN prepared from the aqueous gel of 20% w/w PL. The loading of both molecules was 20% w/w in the dry state of PL. Both molecules were detected in the receiver compartment of the Franz cells as soon as 15 min after application of DMN arrays. Following application of the PL DMN for 28 h, 1479 ± 364 μg/cm^2^ of Flu-Na and 1105 ± 123 μg/cm^2^ of FITC-BSA were delivered from PL DMN arrays. As shown, BSA-FITC is a large protein with a molecular weight of 66 kDa, and its release efficiency is lower compared to Flu—Na. It is agreed that the delivered dose from MNs is typically restricted to between the microgram to lower milligram range. The deliverable dose from DMN is defined by the total array volume (i.e. shape, size, and density of MNs) and percentage loading of the drug [26]. The appliance of biomolecules delivery *using* dissolving MNs outcomes in polymer deposition in the skin. As most of the drug and biomolecule delivery regimens require repeated application of dissolving MNs, regulatory bodies will need assertion of polymer safety. Accordingly, a considerate of the long-term effects of polymer deposition will necessitate further investigation to ensure they do not represent a toxicity issue. While dissolving MNs are typically fabricated from high molecular weight synthetic polymers that not necessarily biocompatible. As highlighted such polymer deposition may result in effects such as erythema or hepatic/lymphatic accumulation and require further investigation [27]. The main advantage of PL for MN manufacture is that PL is inherently biocompatible and this is widely discussed in the literature. The biocompatibility of PL has significant positive implication for repeat application of DMN manufactured from PL, particularly in delivery of drug/biomolecules [19,32,33]. Therefore, PL based DMN will not have significant long-term secondary/detrimental effects. This novel biopolymer PL based DMN array could be used for the transdermal delivery of small molecule drugs and large biomolecules up to a low milligram dose. This preliminary formulation work using PL biopolymer supports the growing evidence surrounding pain and needle-free, self-administered, transdermal delivery systems. Further pre-clinical work is required to assess the long term efficacy and tolerability of PL-based DMN for transdermal delivery.

## 4 Conclusion

The work presented here illustrates the potential of carbohydrate biopolymer PL-based DMN to deliver low and high molecular weight drug molecules across the skin. These first time fabricated DMN were sufficiently robust to penetrate the skin and dissolve rapidly in the skin to achieve effective drug permeation. PL DMN arrays were successfully formulated with model dyes, MB and Flu-Na as well as model protein/peptide, FITC-BSA and insulin. The stability of model compounds, FITC-BSA and insulin, in PL DMN arrays was confirmed by circular dichroism. PL DMN successfully managed to deliver the model compounds across excised neonatal porcine skin. Therefore, these self-dissolving microneedles fabricated from biopolymer PL with good mechanical strength and biocompatibility make a viable alternative for enhanced transdermal drug delivery. PL DMN can be developed as a pain-free alternative to traditional needle-based administration for small and large biomolecules.

## CRediT authorship contribution statement

**Lalitkumar K. Vora:** Conceptualization, Methodology, Writing - original draft. **Aaron J. Courtenay:** Visualization, Investigation, Data curation, Writing - review & editing. **Ismaiel A. Tekko:** Visualization, Investigation, Writing - review & editing. **Eneko Larrañeta:** Data curation, Software, Validation, Writing - review & editing. **Ryan F. Donnelly:** Data curation, Funding acquisition, Writing - review & editing, Supervision.
